# Partially substituting chemical NPK fertilizers and their impact on Eureka lemon trees (*Citrus limon* L. Burm) productivity and fruit quality

**DOI:** 10.1038/s41598-023-37457-7

**Published:** 2023-06-28

**Authors:** Abdulrhman A. Almadiy, Ayman E. Shaban, Ammar M. Ibrahim, Salem M. Balhareth, Sherif F. El-Gioushy, El-Sayed G. Khater

**Affiliations:** 1 Biology Department, Science and Art College, Najran University, Najran, Saudi Arabia; 2 Pomology Department, Faculty of Agriculture, Cairo University, Giza, 12511 Egypt; 3 Ministry of Environment and Water and Agriculture, Najran Horticulture Research Centre, Najran, Saudi Arabia; 4 Horticulture Department, Faculty of Agriculture, Benha University, Moshtohor, P.O. Box 13736, Toukh, Kalubia Egypt; 5 Agricultural and Biosystems Engineering Department, Faculty of Agriculture, Benha University, Moshtohor, P.O. Box 13736, Toukh, Kalubia Egypt

**Keywords:** Ecology, Environmental sciences

## Abstract

The main aim of this study is to improve productivity, physical and chemical properties of the fruits and fruit quality of Eureka Lemon trees while lowering production costs by investigating the use of different NPK alternative sources (slow release, and bio) to reduce the use of chemical NPK fertilizers. Ten treatments of NPK fertilizers were applied. The results indicate that the highest values of yield (111.0 and 114.0 kg/tree) were found with 100% chemical NPK (control) for both first and second seasons, respectively. The lemon fruit weight ranged from 131.3 to 152.4 and 131.4 to 153.5 g for first and second seasons, respectively, for all treatments under study. The highest values of fruit length and fruit diameter were found with 100% chemical NPK (control) for both two seasons. The highest values of juice quality parameters (TSS, juice acidity, TSS/acid ratio and vitamin C concentration) responded favorably to higher chemical NPK treatment rates. The highest values of TSS, juice acidity, TSS/Acid ratio, and vitamin C concentration were 9.45%, 6.25%, 1.524 and 4.27 mg/100 g, respectively, were found with 100% chemical NPK (control) for both two seasons. Meanwhile, the lowest value of total sugar was found with 100% chemical NPK (control) for both two seasons.

## Introduction

Eureka lemon is considered a popular type of lemon and is characterized by yellow fruits and greenish yellow pulp. It is sensitive to low temperature conditions. Their tree reaches 20 feet height. Their fruits are oval in shape and its colour from green to yellow when ripening. It contains acidic juice in its pulp that which is divided into 8–10 segments. Their fruits contains: peel (exocarp) which is thick outer shell, albedo (mesocarp) is a while spongy part, this is rich in pectin. Endo carp contains the juice and contains organic acid and sugar with water and acidic in taste. Some fruits have small oval white or yellowish-white seeds^1^. Lemon (*Citrus limon* L.) ranked as the third major grown citrus species after both orange and mandarin belongs^2,3^. It belongs to Rutaceae family^4^. The total lemon cultivated area in Najran city is approximately 9000 Dunum producing about 20,599 tons fruits per year with an average about 2.29 tons fruits per Dunum. The total production of lemon fruits in Saudi Aribia country about 51,000 ton fruits per year according to^3^.

The fruits contains natural flavor and preservative added to various foods and salad, sauces, and baked foods. Lemon juice is used as soft drinks and the addition food products to give acidic taste^5^. Lemon juice is an important source of vitamin C, which is good for immunity of the human body. Also, it is rich with flavonoids, antioxidants, which is useful in removing the free radicals that harm the tissue cells in the body, eating foods containing flavonoid protect from cancer and cardiovascular diseases^6^.

Nitrogen (N) is very important as mineral fertilizers for citrus and affects the growth, yield and quality of fruits. Potassium is effective in physiological functions like translocation of sugars, synthesis of proteins and cell division and growth. It is very useful in fruit growth and improves the size, flavor and color of fruits. Phosphorus is required in photosynthesis, synthesis and carbohydrates breakdown and energy transfer within plant. These days, chemical fertilizers are important in fruit crop nutrition, but using it in an excessive quantity has bad effect on soil, water and atmosphere which in turn animal and human health. It had also effect on soil fertility, water quality, yield and products quality^7–9^.

Therefore, organic manure and biofertilizers have been used for sustainable production, which improve soil physical, chemical and biological properties. Many researchers reviewed the significant role of organic and biofertilizers in improving the soil and plant properties for crops such as Sweet orange^10,11^; on lemon^12–14^.

Soil microbes are necessary in several ecosystem processes such as nutrient cycling and decomposition of organic matter, as well as enhancing plant health and growth^15–19^.

Regarding that, the goal of this study was to look into the possibility of lowering the high cost of chemical fertilizers (NPK), which have a direct impact on human health, by using less expensive alternatives that are also environmentally friendly, such as (chemical, slow release, and bio), and their effect on the productivity and fruit quality of Eureka Lemon Trees (*Citrus limon* L. Burm).

## Materials and methods

The present investigation was undertaken during the two consecutive seasons of 2021 and 2022 in a private orchard at Nagran region, Saudia Arabia (latitude 17° 36′ N and 44° 26′ E) on 21 years old Eureka lemon trees. Thirty healthy fruitful Eureka lemon trees budded on sour orange (*Citrus aurantium *L.) rootstock, were carefully selected and devoted for achieving this study. The selected trees were nearly uniform as possible as we could in their growth vigour, free from diseases, grown in clay loamy soil and planted at 4 × 5 m (210 trees/feddan) apart under drip irrigation system. Citrus fruits have been collected under the permission of Nagran University regulations. Plant materials are complied with the local and national regulations. The average temperature of 24.6 ± 5.3 °C and relative humidity of 73.2 ± 6.7%.

The soil texture in this trial was clay loamy textured. Moreover, mechanical and chemical analysis of the experimental soil is shown in Table 1. The results of soil and farmyard manure analysis according to^20^ are given in Table 1. All chosen trees received the recommended agriculture practices except fertilization. Thirty trees were arranged in a randomized complete block design, each treatment replicated three times with three trees per replicate.

**Table 1:** Some physical and chemical analysis of the experimental sandy soil used.

Sand (%)	Silt (%)	Clay (%)	Texture	pH 01:02.5	E.C. (dS/m) 1:5	TDS (ppm)	N (%)	P (%)	K (%)	Ca (%)	Fe (ppm)	Mn (ppm)	Zn (ppm)
78.9	12.5	8.6	Loamy sand	7.56	1.28	819	2.3	0.11	0.46	5.36	61	29	15

Accordingly the investigated NPK sources and bio-fertilizers soil applied fertilization treatments were as follows:

**Table:** 

Without-Bio NPK	With-Bio NPK
T1: Control (100% chemical NPK)	T6: Control (100% chemical NPK)
T2: 75% chemical NPK + 25% natural sources	T7: 75% chemical NPK + 25% natural sources
T3: 50% chemical NPK + 50% natural sources	T8: 50% chemical NPK + 50% natural sources
T4: 25% chemical NPK + 75% natural sources	T9: 25% chemical NPK + 75% natural sources
T5: 100% natural sources	T10: 100% natural sources

Rate and application method of chemical NPK fertilizers.

Four rates of chemical fertilizers NPK were employed in this study. The first rate was 100% of NPK (1000:250:500 g/tree/year, respectively) were applied as 4.85 kg/tree ammonium sulphate (20.6% N), 1.60 kg/tree super phosphate (15.5% P_2_O_5_) and 1.00 kg/tree potassium sulphate (48% K_2_O). The second rate was 75% of NPK (750, 187.5 and 375 g per tree, respectively). The third rate was 50% of NPK (500, 125 and 250 g per tree, respectively). The fourth rate was 25% of NPK (250, 62.5 and 1255 g per tree, respectively). Nitrogen fertilizer was added on three doses, at March, the first of June and at the end of August. Whereas, potassium was applied on two doses, at the first of March and at the end of August with nitrogen fertilization.

Rate and application method of Natural alternative NPK fertilizations mixture (organic N and NPK raw mineral rocky materials).

However, other investigated alternate NPK fertilizers sources were: 1—granulated organic N fertilizer of 18–20% actual N, 2—two natural raw rocky materials, 1st as P fertilizer of 18–20% actual P_2_O_5_*, while 2nd as K fertilizer of 10–12% actual K_2_O. Were used in four rates (the first rate was 100% of alternate NPK (10:1.250:4.17 kg/tree/year, respectively). The second rate was 75% of alternate NPK (7.5, 0.937 and 3.13 g per tree, respectively). The third rate was 50% of alternate NPK (5, 0.625 and 2.08 kg per tree, respectively). The fourth rate was 25% of alternate NPK (2.5, 0.312 and 1.04 kg per tree, respectively). Were applied in soil (15 cm depth) in one dose in early December.

Rate and application Method of Bio-organic fertilizers:

A mixture of three types of bio-fertilizers (equal amounts for each) was investigated throughout this study. These types namely:

Phosphorene: a commercial phosphor bio-fertilizer, which contains some active fungi strains (Arbuscalar mycorrhiza).

Nitrobein: a commercial nitrogen bio-fertilizer contains special bacteria (Azotobacter choroccocum).

Potassein: a commercial potassium bio-fertilizer contains special bacteria (Bacillus pasteurii).

Each of the three bio-fertilizers mentioned above, were applied in soil (15 cm depth) to the wetted soil in one dose in early December at a rate of 50 ml per tree. These treatments were replicate three times.

### Measurements

#### Yield per tree

At harvest time (first week of December in both seasons); the fruit yield of each tree was recorded as weight (kg).

#### Fruit quality measurements

In first week of December in both seasons, which is the harvest time, ten fruits from each tree or replicate were chosen randomly and taken to the laboratory to estimate their physical and chemical fruit characteristics. Fruit weight (g) was calculated by recording the average weight of 10 fruits from each tree/replicate. Average fruit length (L) and fruit diameter (D) were measured using a hand caliper. Fruit volume (cm^3^) was calculated by dipping the fruit in water and weighing the removed water. Also, the weight of the peel (g) and flesh (g) was estimated by electric digital balance (Model Vibra-Range 0–12,000 g ± 0.01 g, Japan). A Magness and Taylor pressure tester measured fruit firmness (Ib/in.^2^) with a 7/18-in. plunger.

The total soluble solids percent (T.S.S.%) was measured by using a hand refractometer (ATAGO Co., LTD., Tokyo, Japan) on the fresh-cut lemon fruit, and the result was expressed as a percentage (%). Total and reducing sugars were estimated calorimetrically using the Nelson arsenate–molybdate colorimetric method^21^. Non-reducing sugars were measured by the difference between total sugars and reducing sugars. The percentage of titratable acidity in fruit juice was determined using an A.O.A.C. method^22^, where it was expressed as g citric acid/100 ml of fruit juice. The TSS/acid ratio was calculated by dividing the value of TSS over the value of titratable acidity. Ascorbic acid (vitamin C) content of the juice was estimated by titration with 2,6-dichloro phenol-indo-phenol^23^ and calculated as mg/100 mg of juice.

All methods used in this study were carried out according to the guidelines regulations of Benha and Nagran Universities.

### Statistical analysis

All the obtained data in the two seasons of study were statistically analyzed using the analysis of variance method according to^24^. However, means were distinguished by the Duncan’s multiple range test^25^. Since, capital letters were used for distinguishing means within each column or row that represented the specific effect of any investigated factor (NPK) applied level and some bio-fertilizers soil added however, the small letters were employed for interaction effect of their combinations.

## Results

### Yield per tree (kg)

**Figure 1:**
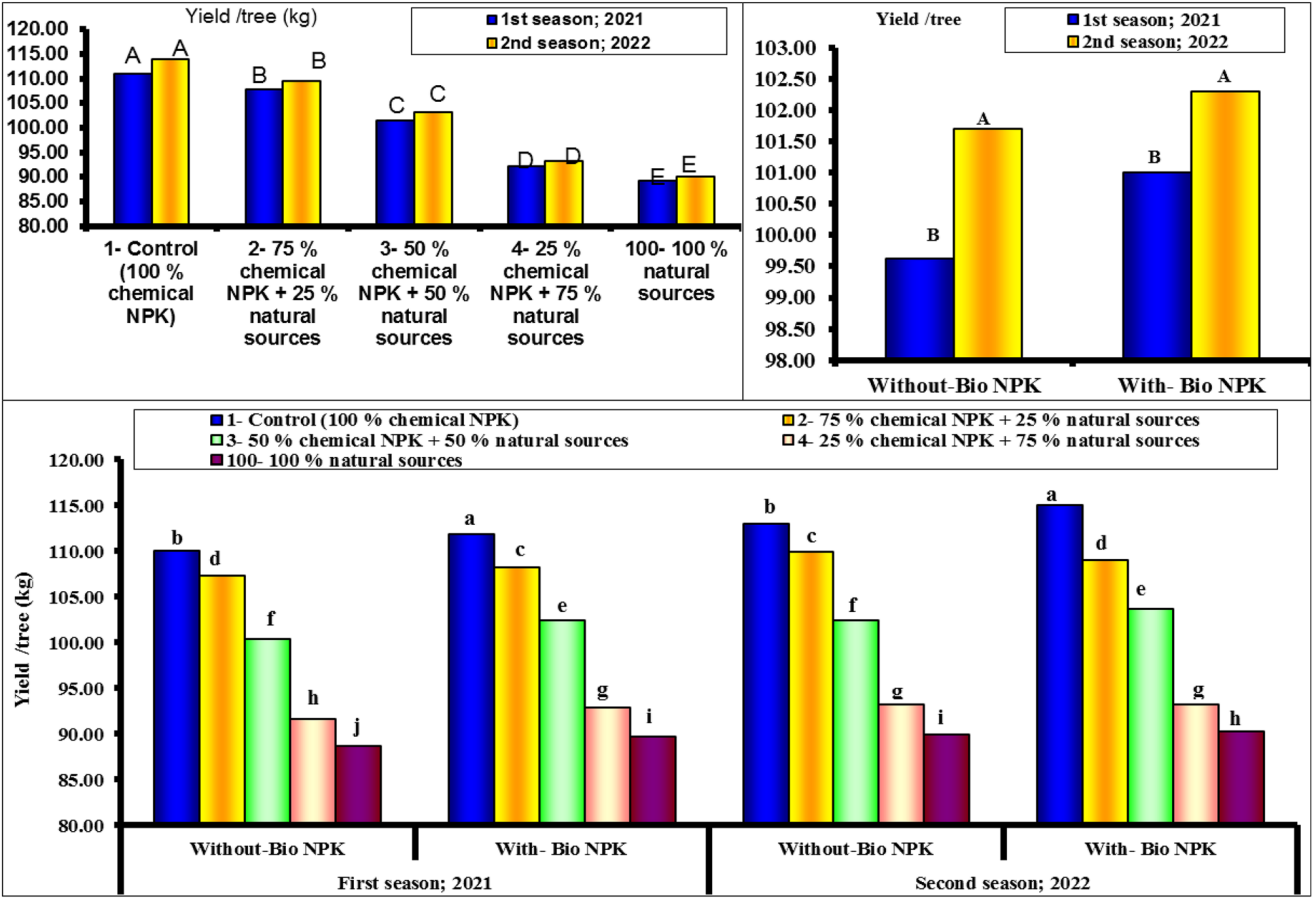
Effect of different NPK sources on yield of Eureka lemon trees during the experimental seasons of 2021 and 2022.

Figure 1 shows the yield of lemon as affected by different NPK sources as replacement for chemical NPK. It could be seen that, adding bio-NPK had a positive influence on yield per tree in both seasons (kg). Increasing the rates of chemical NPK application also resulted in consistently significant increases in the values recorded for the yield per tree. As a result, the highest values were obtained during both studies seasons when the highest levels of chemical NPK (100 and 75%) were combined with bio-NPK.2.5. It could be seen that the highest values of yield (111.0 and 114.0 kg/tree) were found with 100% chemical NPK (control) for both first and second seasons, respectively. While, the lowest values of yield (89.75 and 90.34 kg/tree) were found with 100% natural sources for both first and second seasons, respectively.

### Physical properties of the product

#### Fruit weight and fruit size

**Figure 2:**
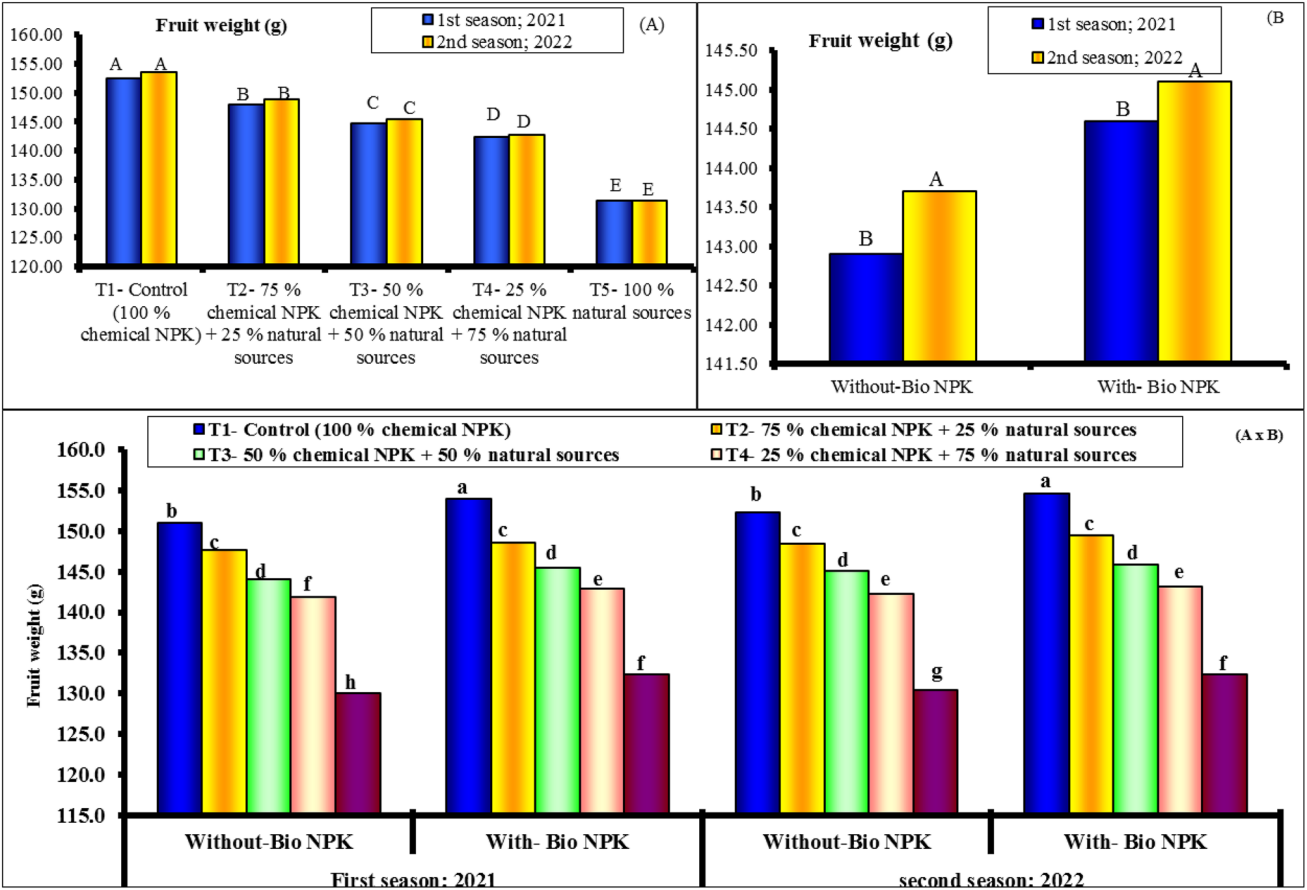
Effect of different NPK sources on fruit weight of Eureka lemon trees during the experimental seasons of 2021 and 2022.

**Figure 3:**
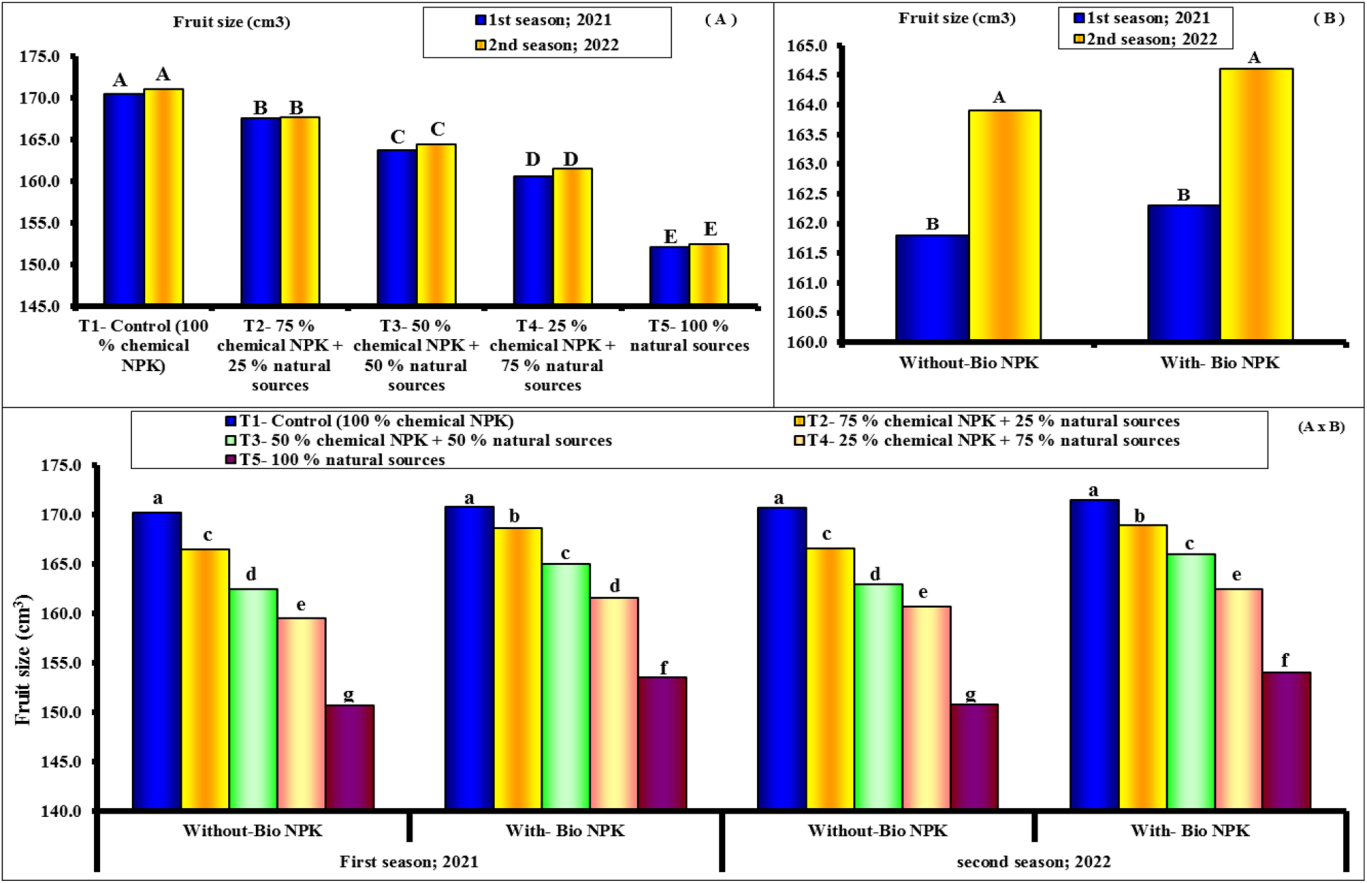
Effect of different NPK sources on fruit size of Eureka lemon trees during the experimental seasons of 2021 and 2022.

Fruit weight (g) and fruit size (cm^3^) of Eureka lemon trees were influenced by different levels of minerals NPK and their combinations with bio-NPK or without bio-NPK during the 2021 and 2022 seasons, according to data presented in Figs. 2 and 3. The results indicate that the fruit weight was 152.4, 148.0, 144.7, 142.4 and 131.3 g for 100% chemical NPK (control), 75% chemical NPK + 25% natural sources, 50% chemical NPK (control), 50% chemical NPK + 25% natural sources, 50% chemical NPK (control), 25% chemical NPK + 75% natural sources and 100% natural sources, respectively, for first season. Also, it was 153.5, 148.9, 145.5, 142.8 and 131.4 g for 100% chemical NPK (control), 75% chemical NPK + 25% natural sources, 50% chemical NPK (control), 50% chemical NPK + 25% natural sources, 50% chemical NPK (control), 25% chemical NPK + 75% natural sources and 100% natural sources, respectively, for second season.

In terms of the effect of different NPK sources, data obtained during both seasons revealed that when compared to other treatments, all NPK levels and sources increased significantly in all investigated parameters. This was true throughout both seasons. Furthermore, in terms of fruit weight and size, the most effective levels were significantly associated with its highest levels (100% chemical NPK). The data presented in Figs. 2 and 3 revealed that the rate of response to bio-NPK was relatively pronounced with the different NPK sources. The highest levels of minerals NPK (100 and 75%) resulted in a slight increase in fruit weight and size when combined with bio-NPK. The highest values of fruit size (170.5 and 171.1 cm^3^) were found with 100% chemical NPK (control) for both first and second seasons, respectively. While, the lowest values of yield (152.1 and 152.4 cm^3^) were found with 100% natural sources for both first and second seasons, respectively.

#### Fruit dimensions and fruit shape index

**Figure 4:**
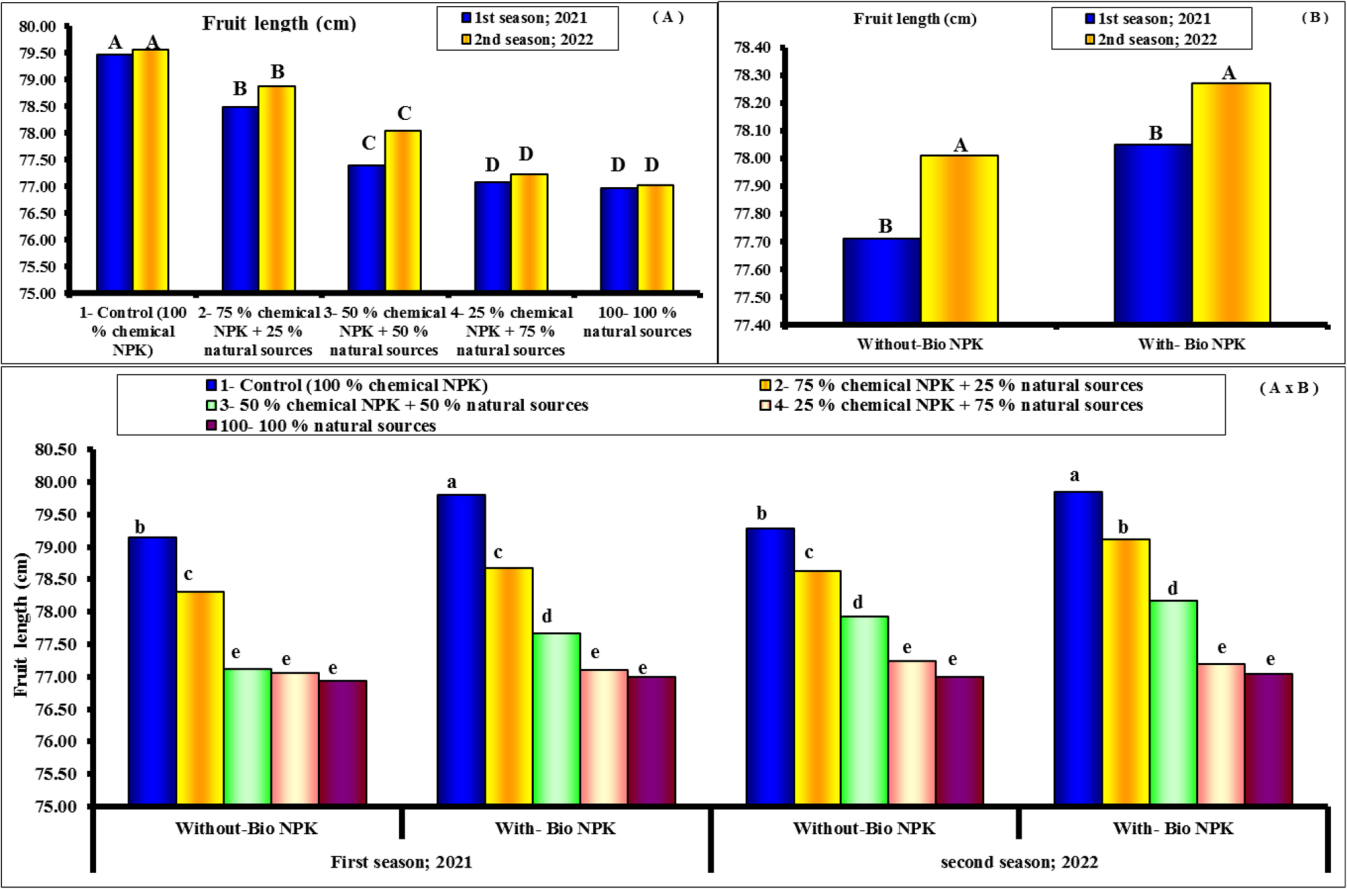
Effect of different NPK sources on fruit length of Eureka lemon trees during the experimental seasons of 2021 and 2022.

**Figure 5:**
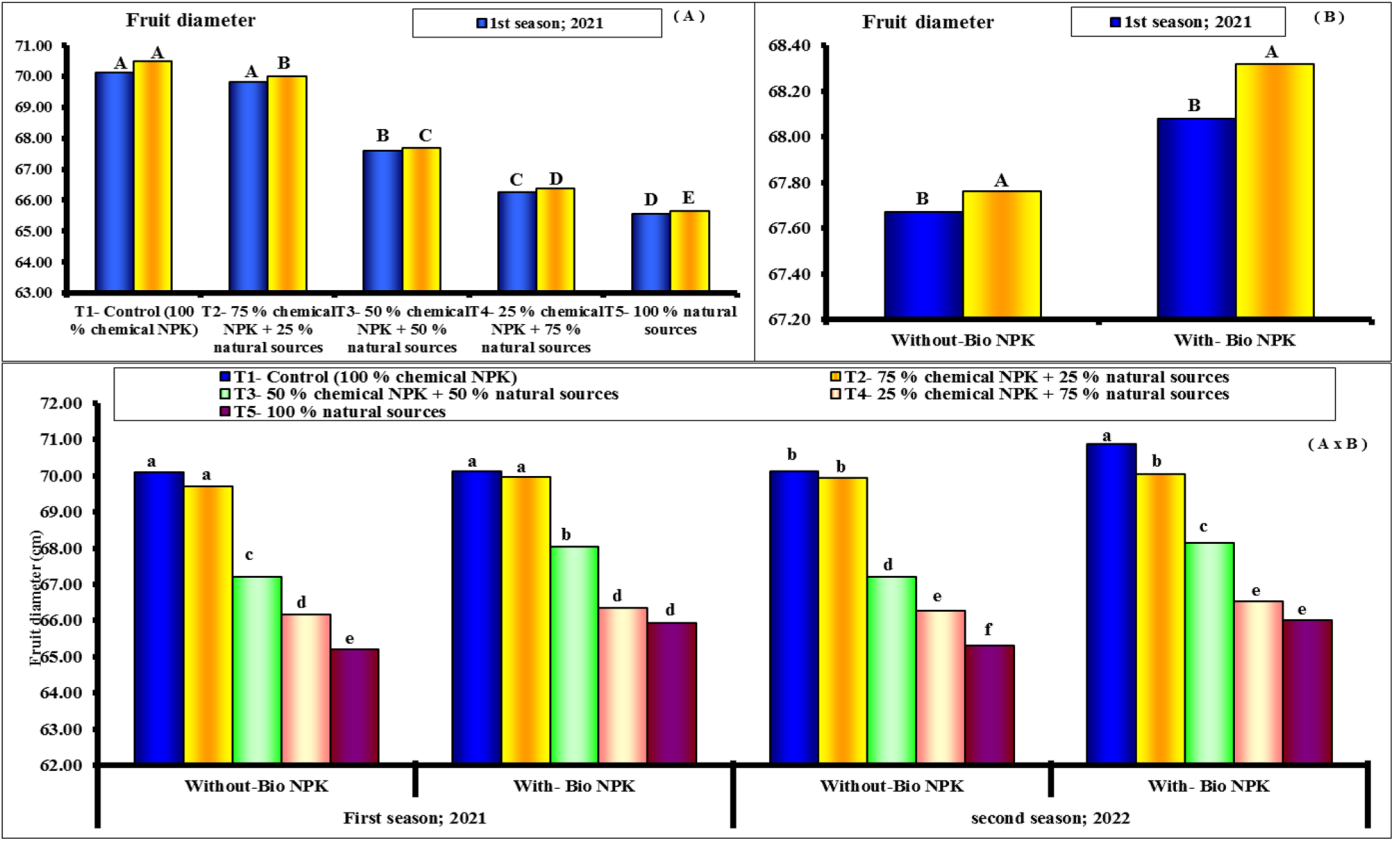
Effect of different NPK sources on fruit diameter of Eureka lemon trees during the experimental seasons of 2021 and 2022.

**Figure 6:**
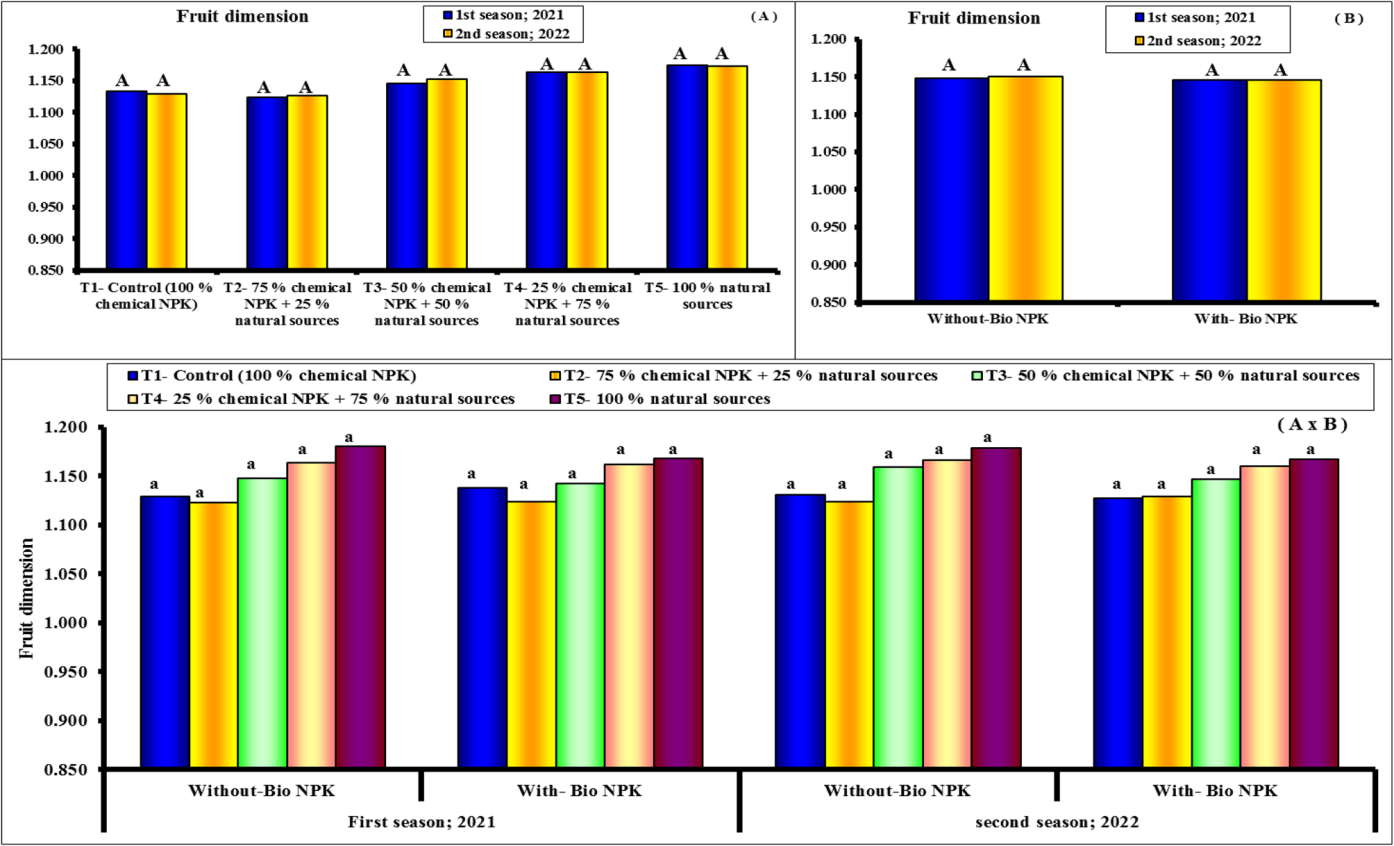
Effect of different NPK sources on fruit dimension (F.L./F.D.) of Eureka lemon trees during the experimental seasons of 2021 and 2022.

Figures 4, 5 and 6 show the length and fruit diameter of Eureka lemon fruits were the investigated two fruit dimensions regarding their response to the differential nutritive compounds. The results show obviously that both parameters responded significantly to all treatments. However, rate of response was relatively higher with former fruit dimension (length) than other one (diameter). Moreover, first treatment (100% chemical NPK + bio-NPK) was the superior and resulted significantly in the tallest length and diameter. Statistically followed by the 2nd treatment 100% chemical NPK + bio-NPK). Such trend was true during both experimental seasons for both length and fruit diameter. The results indicate that, the variability in the fruit shape index (length: diameter) of Eureka lemon fruits in response to the various tested treatments were relatively too small to be taken into consideration statistically. Herein, it can be stated that during both experimental seasons, fruits that had been treated with fertilizers, whether separately or in combination with bio-NPK, tended to be slightly oblong in shape. The highest values of fruit length and fruit diameter (79.47 and 70.11 cm) were found with 100% chemical NPK (control) for first season. While, the lowest values of fruit length and fruit diameter (76.96 and 65.56 cm) were found with 100% natural sources for first season. Also, the highest values of fruit length and fruit diameter (79.56 and 70.50 cm) were found with 100% chemical NPK (control) for second season. While, the lowest values of fruit length and fruit diameter (77.02 and 66.00 cm) were found with 100% natural sources for second season.

#### Flesh weight, fruit firmness and juice

**Figure 7:**
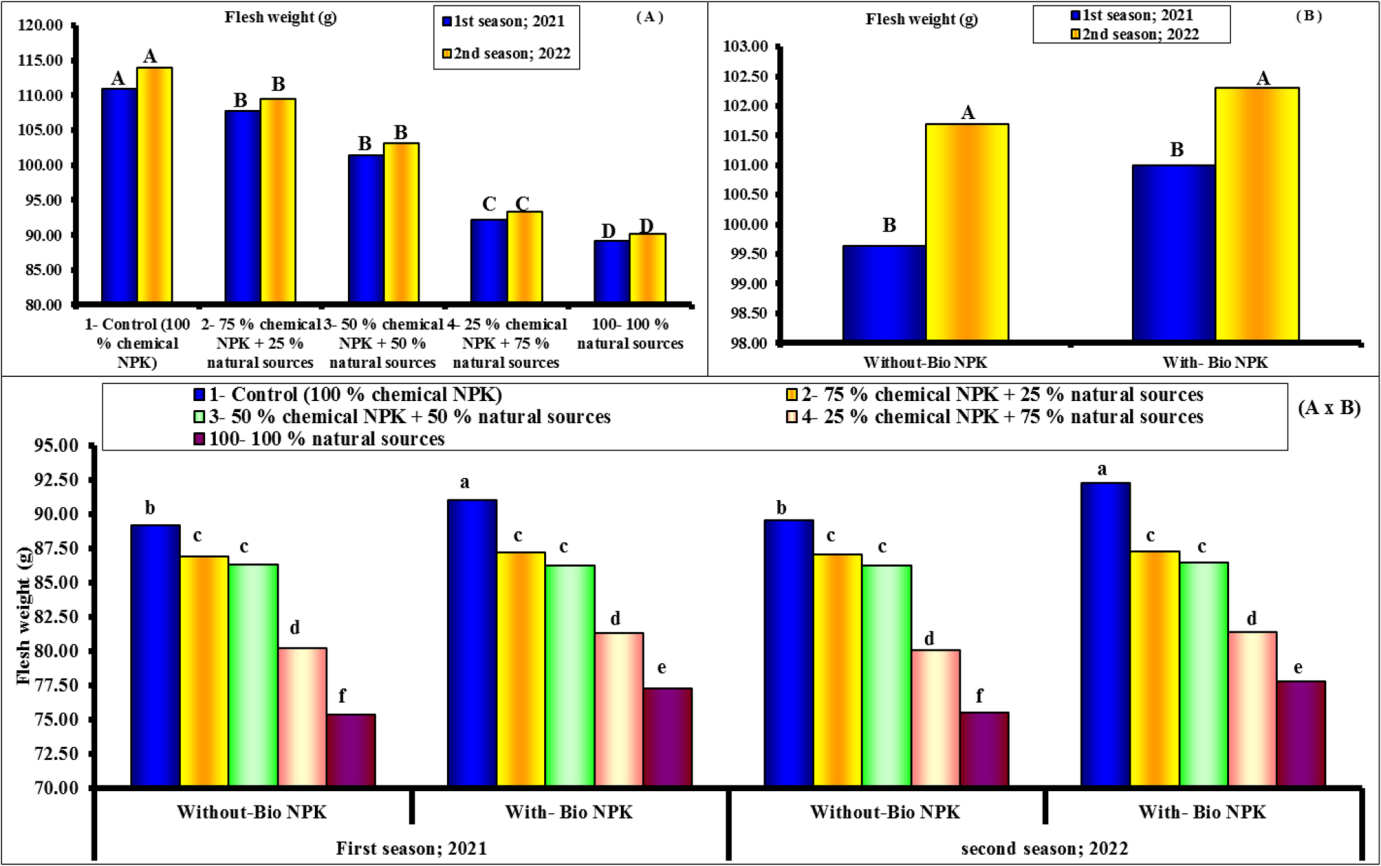
Effect of different NPK sources on flesh weight of Eureka lemon fruits during the experimental seasons of 2021 and 2022.

**Figure 8:**
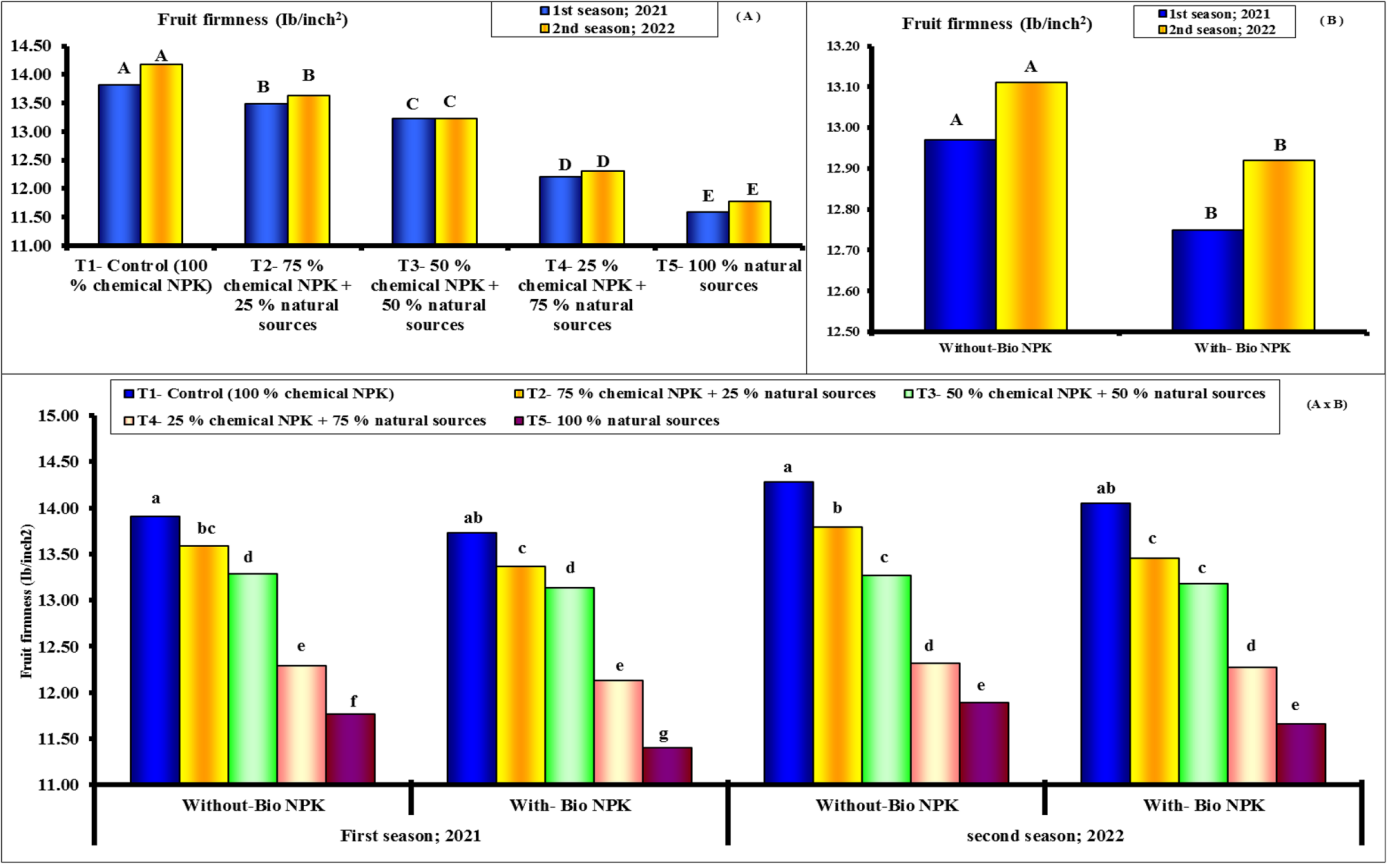
Effect of different NPK sources on fruit firmness of Eureka lemon fruits during the experimental seasons of 2021 and 2022.

**Figure 9:**
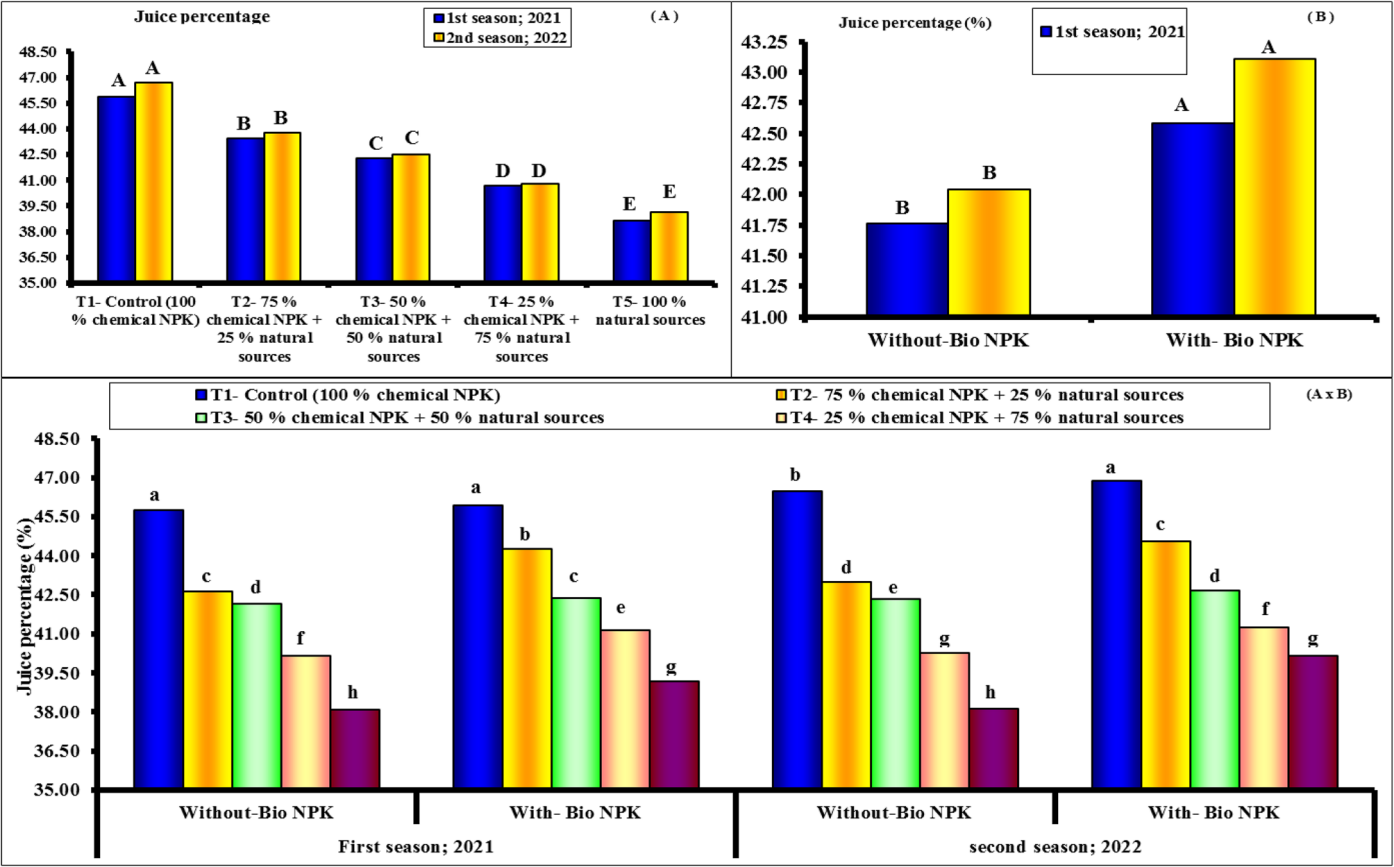
Effect of different NPK sources on Juice percentage of Eureka lemon fruits during the experimental seasons of 2021 and 2022.

Figures 7, 8 and 9 demonstrate unequivocally that the various NPK sources treatments had a positive impact on the recorded flesh weight (g), fruit firmness (lb/in.^2^) and juice (%) in both seasons. Additionally, by increasing the application rates of chemical NPK, the values recorded for the various above-mentioned parameters (Flesh weight and juice %) showed consistent considerable increases. Accordingly, the highest values for the different parameters were recorded when the highest level of chemical NPK (100 and 75%) was combined with bio-NPK during both seasons of study. On the contrary, the least values were significantly in concomitant to the 100% nature sources of NPK during both seasons. As application rates of chemical NPK were increased, fruit hardness gradually increased significantly. Accordingly, the maximum values for the various fruit's firmness were noted during both study seasons when the highest level of chemical NPK (100 and 75%) was combined without bio-NPK. The highest values of flesh weight, fruit firmness and juice percentage (90.10 g, 13.82 lb/in.^2^ and 45.85%) were found with 100% chemical NPK (control) for first season. While, the lowest values of flesh weight, fruit firmness and juice percentage (76.32 g, 11.59 lb/in.^2^ and 38.63%) were found with 100% natural sources for first season. Also, the highest values of flesh weight, fruit firmness and juice percentage (90.91 g, 14.17 lb/in.^2^ and 46.69%) were found with 100% chemical NPK (control) for second season. While, the lowest values of flesh weight, fruit firmness and juice percentage (76.67 g, 11.77 lb/in.^2^ and 39.14%) were found with 100% natural sources for second season.

### Fruit chemical properties

The five tested fruit juice chemical characteristics for Eureka lemon fruits regarding their response to the evaluated treatments were total soluble solids (TSS)%, total acidity%, TSS/acid ratio, total sugars%, and ascorbic acid (vitamin C) contents. Tables 2 and 3 display the data obtained during the experimental seasons of 2021 and 2022. The results indicate that, the majority of the measured juice quality parameters (TSS%, juice acidity%, TSS/acid ratio, total sugars%, and vitamin C concentration) responded favorably to higher chemical NPK treatment rates. With the highest application amounts of the chemical NPK (100 and 75%) with bio-NPK, the highest values for these characteristics were obtained in both seasons. However, during both experimental seasons, the lowest results were found when the highest concentrations of natural sources (100 and 75%) were used independently or in combination with bio-NPK. The highest values of TSS (9.41 and 9.46%) were found with 100% chemical NPK (control) for both first and second seasons, respectively. While, the lowest values of TSS (7.80 and 7.91%) were found with 100% natural sources for both first and second seasons, respectively. The highest values of acidity (6.17 and 6.25%) were found with 100% chemical NPK (control) for both first and second seasons, respectively. While, the lowest values of acidity (5.61 and 5.60%) were found with 100% natural sources for both first and second seasons, respectively. The highest values of TSS/acidity (1.524 and 1.511) were found with 100% chemical NPK (control) for both first and second seasons, respectively. While, the lowest values of TSS/acidity (1.392 and 1.412) were found with 100% natural sources for both first and second seasons, respectively.

**Table 2:** Effect of different NPK sources on TSS, acidity and TSS/acidity of Eureka lemon fruits during the experimental seasons of 2021 and 2022.

Treatments	TSS (%)			Acidity (%)			TSS/acidity		
	Without-Bio NPK	With-Bio NPK	Mean	Without-Bio NPK	With-Bio NPK	Mean	Without-Bio NPK	With-Bio NPK	Mean
First season; 2020									
1—Control (100% chemical NPK)	9.09b	9.73a	9.41A	6.13a	6.22a	6.17A	1.483b	1.564a	1.524A
2—75% chemical NPK + 25% natural sources	8.31c	8.75b	8.53B	5.82bc	5.88b	5.85B	1.428 cd	1.488b	1.458B
3—50% chemical NPK + 50% natural sources	8.16 cd	8.36c	8.26C	5.72b-e	5.77b-d	5.74C	1.427c	1.449c	1.438BC
4—25% chemical NPK + 75% natural sources	8.03c-e	8.09 cd	8.06C	5.65de	5.70c-e	5.68CD	1.421 cd	1.419 cd	1.420C
100–100% natural sources	7.70e	7.90de	7.80D	5.56e	5.65de	5.61D	1.385e	1.398de	1.392D
Mean	8.25B	8.57A		5.78A	5.84A		1.429B	1.464A	
Second season; 2021									
1—Control (100% chemical NPK)	9.18b	9.73a	9.46A	6.19a	6.32a	6.25A	1.483b	1.540a	1.511A
2—75% chemical NPK + 25% natural sources	8.40c	8.95b	8.68B	5.71bc	5.84b	5.78B	1.471bc	1.533a	1.502A
3—50% chemical NPK + 50% natural sources	8.27 cd	8.49c	8.38C	5.68b-d	5.73bc	5.71BC	1.456b-d	1.482b	1.469B
4—25% chemical NPK + 75% natural sources	8.02de	8.18 cd	8.10D	5.62 cd	5.64 cd	5.63C	1.427de	1.450 cd	1.439C
100–100% natural sources	7.77e	8.04de	7.91D	5.55d	5.65 cd	5.60C	1.400e	1.423e	1.412D
Mean	8.33B	8.68A		5.75B	5.84A		1.447B	1.485A

**Table 3:** Effect of different NPK sources on total sugars and Vitamin C of Eureka lemon fruits during the experimental seasons of 2021 and 2022.

Treatments	Total sugars (%)			Vitamin C		
	Without-Bio NPK	With- Bio NPK	Mean	Without-Bio NPK	With- Bio NPK	Mean
First season; 2020						
1—Control (100% chemical NPK)	1.55 g	1.61f.	1.58E	40.67b	41.87a	41.27A
2—75% chemical NPK + 25% natural sources	1.62ef	1.64e	1.63D	39.36 cd	39.76c	39.56B
3—50% chemical NPK + 50% natural sources	1.68d	1.71d	1.70C	38.31e	38.92d	38.62C
4—25% chemical NPK + 75% natural sources	1.75c	1.78b	1.77B	37.27f.	37.70f.	37.49D
100–100% natural sources	1.78b	1.82a	1.80A	36.30 h	36.77 g	36.54E
Mean	1.68B	1.71A		38.38B	39.01A	
Second season; 2021						
1—Control (100% chemical NPK)	1.61 g	1.64f.	1.62E	41.71b	42.72a	42.21A
2—75% chemical NPK + 25% natural sources	1.70e	1.70e	1.70D	40.20d	40.91c	40.55B
3—50% chemical NPK + 50% natural sources	1.73d	1.74 cd	1.74C	39.36e	39.78de	39.57C
4—25% chemical NPK + 75% natural sources	1.76c	1.82b	1.79B	38.18f.	38.35f.	38.27D
100–100% natural sources	1.82b	1.85a	1.84A	36.83 h	37.50 g	37.17E
Mean	1.73B	1.75A		39.26B	39.85A	

The highest values of total sugars (1.80 and 1.84%) were found with 100% natural sources for both first and second seasons, respectively. While, the lowest values of total sugars (1.58 and 1.64%) were found with 100% chemical NPK (control) for both first and second seasons, respectively. Meanwhile, the highest values of vitamin C (41.27 and 42.21 mg/100 g) were found with 100% chemical NPK (control) for both first and second seasons, respectively. While, the lowest values of vitamin C (36.54 and 37.17 mg/100 g) were found with 100% natural sources for both first and second seasons, respectively.

## Discussion

The results of the effect adding bio-NPK to lemon, where lemon leaf’s SLA, SLW, and RGR were significantly affected by the two biofertilizers treatments with NPK treatments. Al-Freeh et al.^26^ reported that Oat plants treated with biofertilizers gave a higher rate of RGR in the two seasons under study. Also, Kumari et al.^27^ reported that biofertilizers with NPK gave a higher value of RGR in baby corn plants. Biofertilizers work on the development of root structure and increase the flow rate of root xylem as a result of increasing and providing absorption of water and suitable nutrient elements, which leads to a difference in growth rates from plants not treated with biofertilizers^28,29^.

The *T. harzianum* with NPK fertilizer had significantly effect, where it increased the leaf chlorophyll content (44.61%) and chlorophyll fluorescence (F0) (18.36%) over the control plants. Photosynthetic yield (Fv/Fm) and SPAD value of wheat leaves under osmotic stress were increased significantly with the addition of biofertilizers^30^. Mohammed et al.^31^ reported that biofertilizers better affected leaf chlorophyll content on pear trees. Soil inoculation of biofertilizers with chemical fertilizer affects rice growth and yield by increasing leaf area and leaf chlorophyll content^32^. Organic fertilizer application increased chlorophyll content, stomatal conductance, and net photosynthetic rate of pear jujube trees^33^. Osman and El-Rhman^34^ clarified that biofertilizers gave the highest value of leaf total chlorophyll content of fig trees. Biofertilizers produce plant growth hormones (auxin) and organic acids that promote plant growth and enzyme activities and increase chlorophyll content in plant leaves^35^. Arefe et al.^36^ also reported that biofertilizers positively affected chlorophyll content (SAPD value) in basil leaves. In this study, stomatal conductance was positively affected by biofertilizer treatments with NPK. The addition of microbes with inorganic fertilizer may increase the mineral nutrients and water absorptions by improving the root system, thus affecting the leaf stomatal conductance of lemon trees.

Fruit quality characteristics of limau nipis were significantly improved with reduced rates of NPK fertilizers. Our study’s findings agree with the results of^37^, who reported that biofertilizers and farmyard manure with NPK doses improved fruit quality like fruit number, the weight of fruit, and fruit dimension of Eureka lemon trees. Also, Ennab^38^ reported that soil inoculation of beneficial fungi and bacteria improved fruit quality in strawberries. Dheware and Waghmare^10^ mentioned that biofertilizers with NPK increased the number of fruits and weight of fruits in sweet orange. Hadole et al.^19^ reported that the Nagpur mandarin tree was affected by biofertilizers plus NPK, where the yield increased by 50% more than the control treatment. Improved fruit growth and quality could be ascribed to the constant supply of nutrients, especially potassium, higher concentrations of soil enzymes, and growth-promoting substances produced by soil-applied microorganisms, which may have aided in the biosynthesis and translocation of carbohydrates into the fruit^39^. It has been reported that biofertilizers application increased the level of endogenous auxins hormone in treated plants^40^. These elevated levels of auxins in the fruit can promote the sink potential of the fruits, which is positively correlated with the fruit growth rate^41^.

Regarding the soil inoculation of *B. thuringiensis*, it increased the number of fruits (81.81%), fruit weight (55.52%), fruit diameter (43.54%), fruit dimension (35.69%), pulp to peel ratio (94.87%) and fruit juice content (65.36%) compared to the control group. Soil inoculation of Bacillus sppincreases the growth and biomass of roots, shoots, and leaves^42,43^, synthesis of plant growth regulators (IAA, GAs, Cytokinins, and Spermidines)^43,44^, and elevate the levels of photosynthetic pigments, sugars, amino acids, proteins, and mineral nutrients in plants^43,45^ and increase fruit yield^46^. The nitrogenase (nifH) gene of the *Bacillus* spp. produces enzyme nitrogenase which can fix atmospheric N_2_ and supply to plants for stimulation of plant growth and yield^47^. *Bacillus* spp. synthesis plant growth regulators enhance cell division and cell elongation during fruit set and development^43,44^. *Bacillus* spp. also secretes the enzyme ACC deaminase, which inhibits the synthesis of ethylene in plants and enhances the growth of the plants^48,49^. Ismail et al.^50^ reported that fruit quality can be improved only at the production level, but after harvest, the fruits can only maintain their quality. Our results reported that biofertilizers treatment increased the leaf and fruit TSS content limau nipis. Fruit TSS content of Eureka lemon trees is significantly affected by biofertilizers and farm manure which reduce the rate of NPK^37^. It was reported earlier that soil application of biofertilizers increased the fruit TSS content of guava trees^51^. Ye et al.^34^ also stated that applying organic fertilizer improved the total soluble solids content and fruit quality of Jujube. Increasing TSS content in leaves and fruits may be due to improved net photosynthetic rates and plant growth, which increases the accumulation of photosynthates and nutrients, and transfer accumulates to fruits causing improved fruit quality^52^. The solubilization of mineral nutrients, synthesis of plant growth regulators and secondary metabolites, and enzyme secretions from *T. harzianum and B. thuringiensis* confirm their biofertilizer effects on lemon trees towards improving growth physiology, and fruit quality of limau nipis. These results agreed with those obtained by^53–56^.

## Conclusions

This work concluded that adding bio-NPK to lemon tree had great effect on yield and production compared to the chemical NPK, where, the highest values for the various parameters were obtained when the highest level of minerals NPK (100%) was combined with bio-NPK, followed by 75% minerals NPK + 25% of alternative NPK combined with bio-NPK, which could be safely advised given their favourable effects on productivity and fruit quality characteristics. The highest value of yield was 114.0 kg/tree for all treatments under study. The lemon fruit weight ranged from 131.3 to 153.5 g for all treatments under study. The highest values of TSS, juice acidity, TSS/Acid ratio, and vitamin C concentration were 9.45%, 6.25%, 1.524 and 4.27 mg/100 g, respectively, were found with 100% chemical NPK (control) for both two seasons. Future studies are recommended to investigate under range bio-NPK on the storability.

## Data Availability

The datasets used and/or analyzed during the current study available from the corresponding author on reasonable request.

## References

[1] Mabberley DJ (2004). Citrus (Rutaceae): A review of recent advances in etymology, systematics and medical applications. Blumea J. Plant Taxon. Plant Geogr..

[2] Pérez-Jiménez M, Celdrán-Sánchez V, Martínez-Romero D, Pérez-Tornero O (2022). Assessment of the polyamines modulation on cytokinins and ethylene and its effect in lemon (*Citrus limon*) de novo regeneration. Plant Cell Tissue Organ. Cult..

[3] FAO and UNICEF, WFP, WHO (2019). The State of Food Security and Nutrition in the World. Safeguarding Against Economic Slowdowns and Downturns. Rome. Licence: CC BY-NC-SA 3.0 IGO”, 2019.

[4] Rahman M, Islam F, Parvez A, Azad MA, Ashraf GM, Ullah MF (2022). *Citrus limon* L. (lemon) seed extract shows neuro-modulatory activity in an in vivo thiopental-sodium sleep model by reducing the sleep onset and enhancing the sleep duration. J. Integr. Neurosci..

[5] González-Molina E, Domínguez-Perles R, Moreno DA, García-Viguera C (2010). Natural bioactive compounds of *Citrus limon* for food and health. J. Pharm. Biomed. Anal..

[6] Bondonno NP, Dalgaard F, Kyrø C, Murray K, Bondonno CP, Lewis JR, Croft KD, Gislason G, Scalbert A, Cassidy A, Tjønneland A, Overvad K, Hodgson JM (2019). Flavonoid intake is associated with lower mortality in the Danish Diet Cancer and Health Cohort. Nat. Commun..

[7] Srivastava AK, Srivastava AK (2012). Integrated nutrient management. Advances in Citrus Nutrition.

[8] Gupta S, Seth CS (2021). Salicylic acid alleviates chromium (VI) toxicity by restricting its uptake, improving photosynthesis and augmenting antioxidant defense in *Solanum lycopersicum* L.. Phys. Mol. Biol. Plants.

[9] Kumar D, Seth CS (2022). Photosynthesis, lipid peroxidation, and antioxidative responses of *Helianthus annuus* L. against chromium (VI) accumulation. Int. J. Phytoremed..

[10] Dheware RM, Waghmare MS (2009). Influence of organic-inorganic and biofertilizers and their interactions on number of fruits per tree and average weight of fruit of sweet orange (*Citrus sinensis* Osbeck L.). Int. J. Agric. Sci..

[11] Kumar V, Singh MK, Mohan B, Moninder PD (2011). Influence of integrated nutrient management (INM) on yield and quality of Lemon (*Citrus limon* (L.) Burn.) cv. Pant Lemon-1 under Western U.P. conditions. Asian J. Hortic..

[12] Khehra S, Bal JS (2014). Influence of organic and inorganic nutrient sources on growth of lemon (*Citrus limon* (L.) Burm.) cv. Baramasi. J. Exp. Biol. Agric. Sci..

[13] Lal G, Dayal H (2014). Effect of integrated nutrient management on yield and quality of acid lime (*Citrus aurantifolia* Swingle). Afr. J. Agric. Res..

[14] Khehra S, Bal JS (2016). Influence of combined use of organic, inorganic and biological sources of nutrients on fruit quality in lemon. Int. J. Agric. Environ. Biotech..

[15] Hayat R, Ali S, Amara U, Khalid R, Ahmed I (2010). Soil beneficial bacteria and their role in plant growth promotion: A review. Ann. Microbiol..

[16] El-Khayat HM, Abdel-Rehiem MA (2013). Improving mandarin productivity and quality by using mineral and bio-fertilization. Alex. J. Agric. Res..

[17] Khehra S (2014). Improving fruit quality in lemon through INM. Hort. Flora Res. Spectrum.

[18] Babita SS, Ahmed N, Thakur M (2015). Organic farming: A holistic approach towards sustainable fruit production. Eur. J. Pharm. Med. Res..

[19] Hadole SS, Waghmare S, Jadhao SD (2015). Integrated use of organic and inorganic fertilizers with bio-inoculants on yield, soil fertility and quality of Nagpur mandarin (*Citrus reticulate* Blanco). Int. J. Agric. Sci..

[20] Page, A. L., Miller, H. & Keeney, D. R. Methods of soil analysis. Part 2: Chemical and microbiological properties. 2nd Edition, Agronomy Monograph, No. 9, ASA, CSSA, and SSSA, Madison (1982).

[21] Nielsen SS (2010). Phenol-sulfuric acid method for total carbohydrates. Food Analysis Laboratory Manual.

[22] AOAC (2005). Official Methods of Analysis of the Association of Analytical Chemists International Official Methods.

[23] Nielsen SS (2017). Vitamin C determination by indophenol method. Food Analysis Laboratory Manual.

[24] Snedecor GW, Cochran WG (1980). Statistical Methods.

[25] Duncan DB (1955). Multiple range and multiple F tests. Biometrics.

[26] Al-Freeh ML, Alabdulia SA, Huthily KH (2019). Effect of mineral-biofertilizers on physiological parameters and yield of three varieties of oat (*Avena sativa* L.). Basrah J. Agric. Sci..

[27] Kumari E, Sen A, Maurya BR, Sarma BK, Upadhyay PK (2018). Effect of different microbial strains on growth parameters viz. Lai, CGR, RGR and Nar of baby corn. J. Pharmacogn. Phytochem..

[28] Al-Bayati AAK, Al-Joboory JMA, Al-Rawi WMH (2013). Adenisteration of selection Indexes depending on growth parameters and yield components in promising selection genotypes of Barley (*Hordeum vulgare* L.). J. Tikrit Univ. Agric. Sci.ence.

[29] Azarpour I, Moraditochaee M, Bozorgi HR (2014). Effect of nitrogen fertilizer management on growth analysis of rice cultivars. Int. J. Biosci..

[30] Sharifi RS, Khalilzadeh R, Jalilian J (2017). Effects of biofertilizers and cycocel on some physiological and biochemical traits of wheat (*Triticum aestivum* L.) under salinity stress. Arch. Agron. Soil Sci..

[31] Mohammed SM, Fayed TA, Esmail AF, Abdou NA (2010). Growth, nutrient status and yield of Le-Conte pear trees as influenced by some organic and biofertilizers rates with chemical fertilizer. Egypt. J. Agric. Sci..

[32] Naher UA, Qurban AP, Radziah O, Mohd RI, Zulkarmi B (2018). Biofertilizers as a supplement of chemical fertilizer for yield maximization of rice. J. Agric. Food Dev..

[33] Ye S, Liu T, Niu Y (2020). Effects of organic fertilizer on water use, photosynthetic characteristics, and fruit quality of pear jujube in northern Shaanxi. Open Chem..

[34] Osman SM, El-Rhman IE (2010). Effect of organic and bio N-fertilization on growth and productivity on fig trees (*Ficus carica* L.). Res. J. Agric. Biol. Sci..

[35] Panhwar QA, Naher UA, Radziah O, Shamshuddin J, Mohdrazi I, Dipti SS (2015). Quality and antioxidant activity of rice grown on alluvial soil amended with Zn, Cu and Mo. S. Afr. J. Bot..

[36] Arefe R, Ali M, Hassanali N, Farahnaz KH (2013). Effects of bio-stimulators and bio-fertilizers on morphological traits of basil (*Ocimum bacilicum* L.). Ann. Biol. Res..

[37] Ennab HA (2016). Effect of organic manures, biofertilizers and NPK on vegetative growth, yield, fruit quality and soil fertility of Eureka lemon trees (*Citrus limon* (L.) Burm). J. Soil Sci. Agric. Eng. Mansoura Univ..

[38] Todeschini V, Aitlahmidi N, Mazzucco E, Marsano F, Gosetti F, Robotti E, Bona E, Massa N, Bonneau L, Marengo E, Wipf D, Berta G, Lingua G (2018). Impact of beneficial microorganisms on strawberry growth, fruit production, nutritional quality and Volatilome. Front. Plant Sci..

[39] Thejaswini HP, Shivakumar BS, Sarvajna BS, Ganapathi M, Yallesh HS (2022). Studies on split application of NPK fertilizers and liquid bio-formulation (Jeevamrutha) on yield and quality of pomegranate (*Punica granatum* L.) in central dry zone of Karnataka. Pharma Innov. J..

[40] Contreras-Cornejo HA, Macis-Rodriguez L, Alfarocuevas R, Lopez-Bucio J (2014). *Trichoderma* spp. improve growth of Arabidopsis seedlings under salt stress through enhanced root development, osmolite production, and Na+ elimination through root exudates. Mol. Plant Microbe Interact..

[41] Khandaker MM, Boyce AN (2016). Growth, distribution and physiochemical properties of wax apple (*Syzygium samarangense*): A review. Aust. J. Crop Sci..

[42] Ashraf M, Hasnain S, Berge O, Mahmood T (2004). Inoculating wheat seedlings with exopolysaccharide-producing bacteria restricts sodium uptake and stimulates plant growth under salt stress. Biol. Fertil. Soils.

[43] Radhakrishnan R, Lee IJ (2016). Gibberellins producing Bacillus methylotrophicus KE2 supports plant growth and enhances nutritional metabolites and food values of lettuce. Plant Physiol. Biochem..

[44] Xie SS, Wu HJ, Zang HY, Wu LM, Zhu QQ, Gao XW (2014). Plant growth promotion by spermidine-producing Bacillus subtilis OKB105. Mol. Plant Microbe Interact..

[45] Kang SM, Radhakrishnan R, You YH, Joo GJ, Lee IJ, Lee KE, Kim JH (2014). Phosphate solubilizing Bacillus megateriummj1212 regulates endogenous plant carbohydrates and amino acids contents to promote mustard plant growth. Indian J. Microbiol..

[46] Dursun A, Ekinci M, Donmez MF (2010). Effects of foliar application of plant growth promoting bacterium on chemical contents, yield and growth of tomato (*Lycopersicon esculentum* L.) and cucumber (*Cucumis sativus* L.). Pak. J. Bot..

[47] Kuan KB, Othman R, Rahim KA, Shamsuddin ZH (2016). Plant growth-promoting rhizobacteria inoculation to enhance vegetative growth, nitrogen fixation and nitrogen remobilisation of maize under greenhouse conditions. PLoS One.

[48] Xu M, Sheng J, Chen L, Men Y, Gan L, Guo S, Shen L (2014). Bacterial community compositions of tomato (*Lycopersicum esculentum* Mill.) seeds and plant growth promoting activity of ACC deaminase producing *Bacillus subtilis* (HYT-12–1) on tomato seedlings. World J. Microbiol. Biotechnol..

[49] Pourbabaee AA, Bahmani E, Alikhani HA, Emami S (2016). Promotion of wheat growth under salt stress by halotolerant bacteria containing ACC deaminase. J. Agric. Sci. Technol..

[50] Ismail SZ, Khandaker MM, Mat N, Boyce AN (2015). Effects of hydrogen peroxide on growth, development and quality of fruits: A review. J. Agron..

[51] Dutta P, Kundu S, Bauri FK, Talang H, Majumder D (2014). Effect of bio-fertilizers on physico-chemical qualities and leaf mineral composition of guava grown in alluvial zone of West Bengal. J. Crop Weed.

[52] Naik MH, Babu RSH (2007). Feasibility of organic farming in guava (*Psidium guajava* L.). Acta Hortic..

[53] Gupta P, Seth CS (2022). 24-epibrassinolide regulates functional components of nitric oxide signalling and antioxidant defense pathways to alleviate salinity stress in *Brassica juncea* L. cv. Varuna. J. Plant Growth Regul..

[54] Agnihotri A, Seth CS (2016). Exogenously applied nitrate improves the photosynthetic performance and nitrogen metabolism in tomato (*Solanum lycopersicum* L. cv. Pusa Rohini) under arsenic (V) toxicity. Phys. Mol. Biol. Plants.

[55] Yadav M, Gupta PC, Seth CS (2022). Foliar application of α-lipoic acid attenuates cadmium toxicity on photosynthetic pigments and nitrogen metabolism in *Solanum lycopersicum* L.. Acta Phys. Plantarum.

[56] Kumar D, Dhankher OP, Tripathi RD, Seth CS (2023). Titanium dioxide nanoparticles potentially regulate the mechanism (s) for photosynthetic attributes, genotoxicity, antioxidants defense machinery, and phytochelatins synthesis in relation to hexavalent chromium toxicity in *Helianthus annuus* L.. J. Hazard. Mater..

